# Dynamics of PLCγ and Src Family Kinase 1 Interactions during Nuclear Envelope Formation Revealed by FRET-FLIM

**DOI:** 10.1371/journal.pone.0040669

**Published:** 2012-07-24

**Authors:** Richard D. Byrne, Christopher Applebee, Dominic L. Poccia, Banafshé Larijani

**Affiliations:** 1 Cell Biophysics Laboratory, London Research Institute, Cancer Research UK, London, United Kingdom; 2 Department of Biology, Amherst College, Amherst, Massachusetts, United States of America; University of Edinburgh, United Kingdom

## Abstract

The nuclear envelope (NE) breaks down and reforms during each mitotic cycle. A similar process happens to the sperm NE following fertilisation. The formation of the NE in both these circumstances involves endoplasmic reticulum membranes enveloping the chromatin, but PLCγ-dependent membrane fusion events are also essential. Here we demonstrate the activation of PLCγ by a Src family kinase (SFK1) during NE assembly. We show by time-resolved FRET for the first time the direct *in vivo* interaction and temporal regulation of PLCγ and SFK1 in sea urchins. As a prerequisite for protein activation, there is a rapid phosphorylation of PLCγ on its Y783 residue in response to GTP *in vitro*. This phosphorylation is dependent upon SFK activity; thus Y783 phosphorylation and NE assembly are susceptible to SFK inhibition. Y783 phosphorylation is also observed on the surface of the male pronucleus (MPN) *in vivo* during NE formation. Together the corroborative *in vivo* and *in vitro* data demonstrate the phosphorylation and activation of PLCγ by SFK1 during NE assembly. We discuss the potential generality of such a mechanism.

## Introduction

Following fertilisation, the male and female genomes come to occupy a common nuclear compartment, the zygote nucleus [1]. This is accomplished either by fusion of the two pronuclear membranes prior to mitosis or mixture of the parental chromosomes following disassembly of the male and female pronuclear envelopes at prophase and reformation of a common nuclear envelope (NE) after mitosis. These strategies represent respectively the so-called “sea urchin type” and “*Ascaris* type” distinguished by Wilson [2]. In either case, the sperm NE is broken down early in the first cell cycle and a new NE encloses the male genome defining the male pronuclear compartment.

In the sea urchin, once a sperm has entered the egg (which has already completed meiosis) it undergoes a number of transformations before male pronuclear fusion with the female pronucleus [1], [3], [4], [5]. Initially, the sperm mitochondrion and flagellum are lost. Formation of the male pronucleus (MPN) is preceded by vesiculation of the sperm NE which is incapable of typical nucleo-cytoplasmic interactions due to lack of nuclear pores. During vesiculation, sperm chromatin decondenses and a new NE is assembled from membranes largely but not completely derived from the endoplasmic reticulum (ER) of the egg [6], [7]. New pores also assemble. Migration of the MPN and fusion of its outer and inner nuclear membranes with the female pronucleus result in a zygote nucleus.

**Figure 1:**
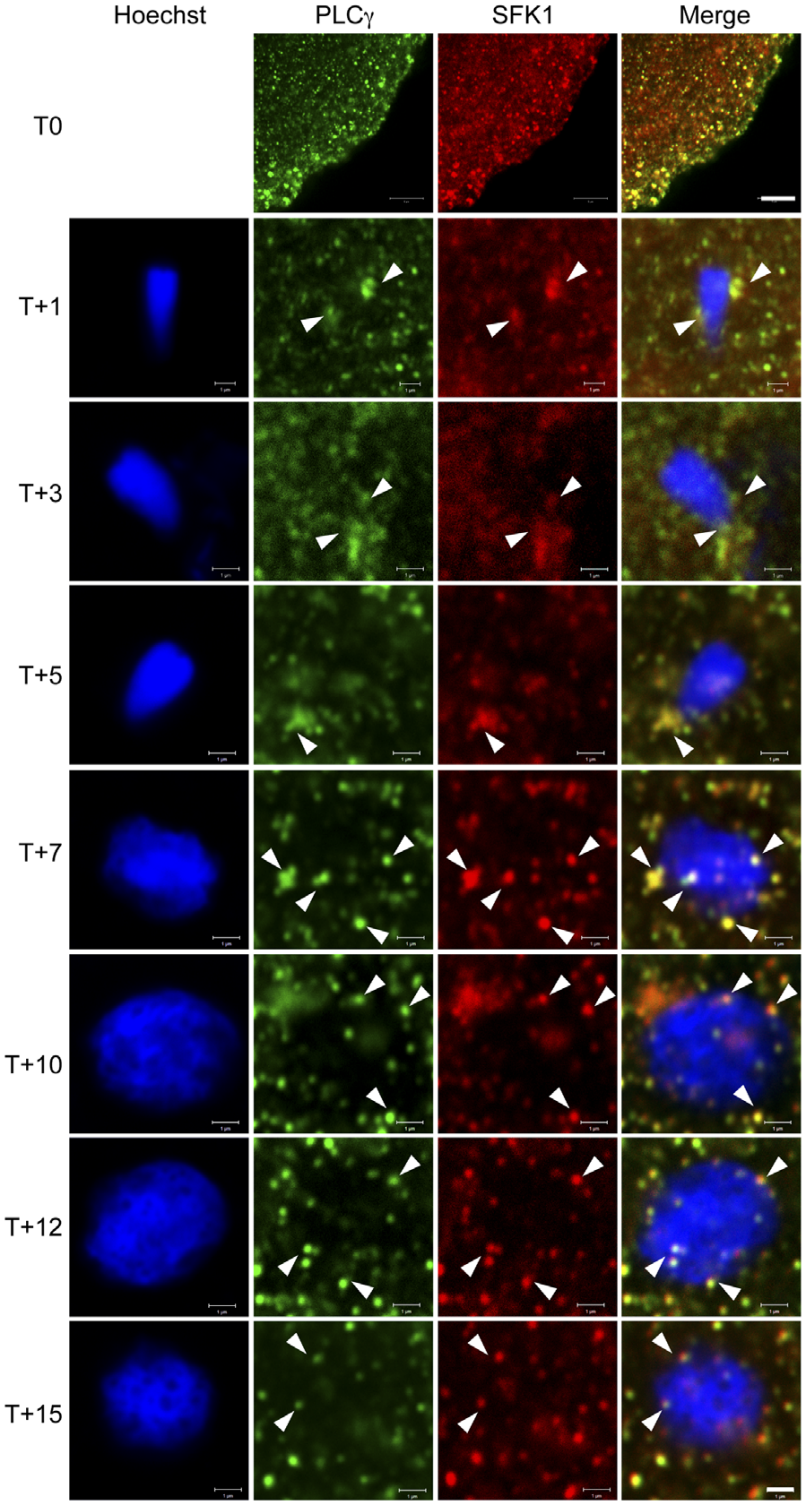
PLCγ and SFK1 are co-recruited to the sperm nucleus *in vivo*. Unfertilised (T0) or fertilised (T+1 onwards) *L. pictus* eggs were fixed and stained with anti-PLCγ (green) and with anti-SFK-Cy5 (red). The sperm nucleus was stained with Hoechst (blue). Arrows (white) indicate vesicles associated with the forming MPN that stain positive for both PLCγ and SFK1. Data show the confocal midsections through nuclei, and are representative of those obtained from three independent experiments. Scale bar is 5 µm (T0) or 1 µm (T+1 onwards).

This process has been detailed morphologically in intact cells by electron microscopy [7], [8]. Extensive biochemical studies on the modification and exchange of sperm and zygotic histone subtypes have been performed on isolated pronuclei [9], [10]. However, elucidation of the biochemical details of MPN envelope reformation has depended on development of a cell-free assay [11]. In this assay, demembranated sperm nuclei are mixed with fertilised egg extracts and, in the presence of the appropriate nucleotides, complete and functional male pronuclear envelopes are formed. While retaining many features of *in vivo* MPN formation, this assay provides a number of experimental advantages: 1) availability of large quantities of synchronous material, making it suitable for analysis with techniques of low read-out sensitivity, 2) ability to manipulate reactions with inhibitors, recombinant proteins and antibodies, and 3) parallel and complementary analyses by traditional biochemical, cell biological and analytical methodologies. Observations from the cell-free assay can be correlated in live sea urchin embryos, which are synchronous and highly suitable for microinjection and microscopy due to their size and transparency [12], [13].

**Table 1:** Summary of FRET experiments *in vivo.*

Time point	FRET efficiency % (egg or MPN ROI)
T0	Egg: 18.9±3.9
T+3	Egg: 5.3±0.8
	MPN: 4.8±0.8
T+5	Egg 2.9±4.2
	MPN: 0.2±6.1

Experiments were performed as described in the methods section and results text. Data show the mean FRET efficiency ± SEM, calculated for each region of interest (ROI), the egg or male pronucleus (MPN).

Our laboratories have described a number of novel aspects of nuclear membrane formation using this assay [14], [15]. These include roles for several membrane populations in forming the new NE including sperm nuclear envelope remnants (NERs) and various egg cytoplasmic membranes [6]. The material provided by the egg includes ER and Golgi membranes, but more strikingly, an additional population of vesicles, termed MV1 [6]. MV1 is highly enriched in phospholipase Cγ (PLCγ) and phosphoinositides, including the PLCγ substrate PtdIns(4,5)P_2_, when compared to the ER membranes that contribute most of the NE [12]. MV1 binds only to the sperm nucleus at the specific polar NER sites, where the remnants of the sperm NE are retained in the acrosomal and centriolar fossae both *in vivo* and *in vitro* [6], [16], [17]. Addition of GTP leads to the transient phosphorylation of PLCγ, a pre-requisite for its activation [12]. Once activated, PLCγ hydrolysis of PtdIns(4,5)P_2_ forms the fusogenic lipid DAG. The localised accumulation of DAG in MV1 leads to NE formation [18], [19], [20]. Initiation of membrane fusion occurs at these sites and subsequently propagates through the ER membranes over the surface of the chromatin to form the fully functional continuous bilayers of the NE [19].

**Figure 2:**
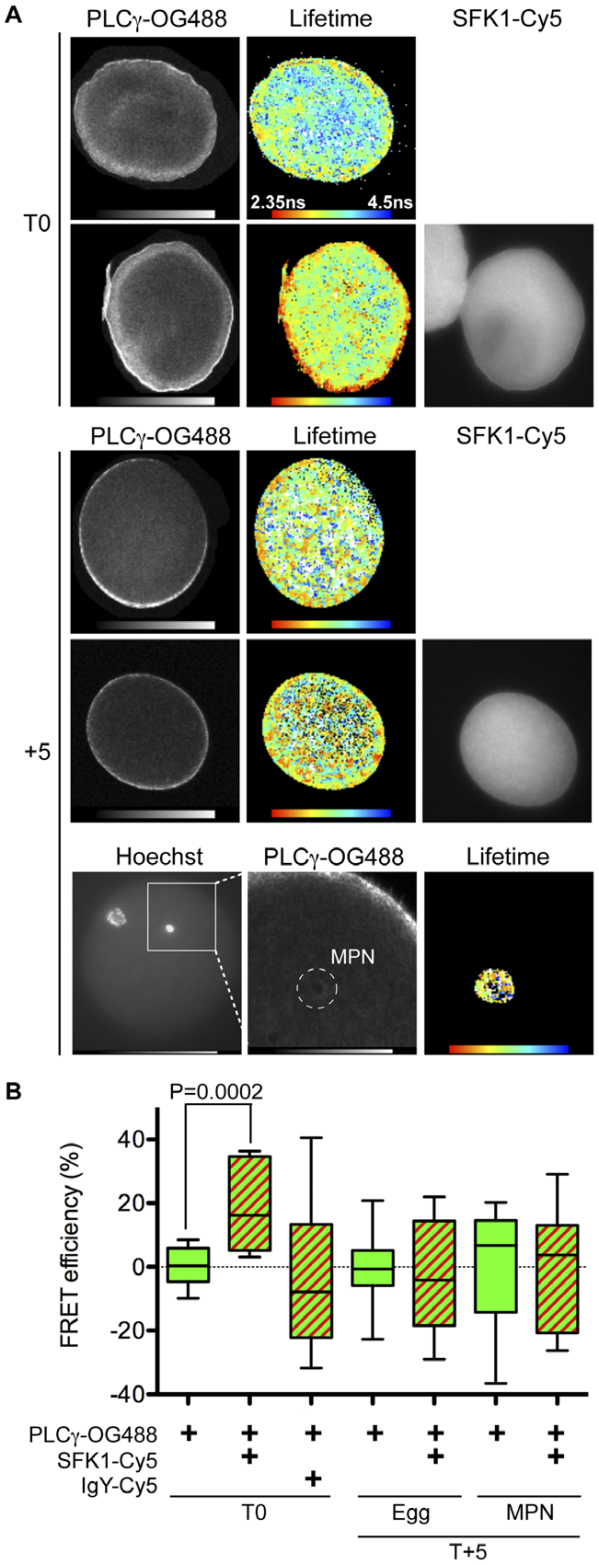
PLCγ and SFK1 interact during the early stages of male pronuclear envelope formation *in vivo*. (**A**) Unfertilised *L. pictus* eggs (T0) or five minutes post-fertilisation (T+5) were fixed and labelled with anti-PLCγ-OG488 alone or together with anti-SFK1-Cy5. Samples were subjected to two-photon time domain FLIM and the lifetime of the donor chromophore (OG488) determined in the absence and presence of acceptor (Cy5). Donor (PLCγ-OG488) two-photon images, donor lifetime ‘heat maps’ and Hg-lamp epifluoresence acceptor (SFK-Cy5) images are shown. At T+5 the donor lifetimes in the whole egg (middle panel) and in the vicinity of the MPN only (bottom panel) were determined. In the donor two-photon image the MPN region of interest is indicated (dashed circle). The donor lifetime heat map and corresponding two-photon image are at the same scale. The images shown are from a donor alone labelled egg. The same process was repeated with eggs labelled with donor and acceptor. Data are representative of at least three independent experiments performed. (**B**) The FRET efficiency for each condition was calculated. Solid green boxes are the donor alone condition, green/red stripes for donor and acceptor conditions. Data are from at least three independent experiments, with a total of 6–18 eggs analysed per condition. Boxes display the median, upper and lower quartiles and whiskers the maximum and minimum values.

The signalling events leading to the activation of PLCγ during NE formation are yet to be elucidated. The PLCγ activation process itself is not fully understood. However it is accepted to involve phosphorylation events, including that of Y783 [21]. More recent findings have enhanced our understanding of the activation process, detailing the auto-inhibitory properties of the PLCγ XY linker region [22], which is mainly mediated by the C-terminus of the two SH2 (cSH2) domains present in the XY linker region. The cSH2 domain was also shown to interact with the Y783 residue when phosphorylated [23]. Thus, phosphorylation of Y783 and its subsequent binding to the cSH2 domain is proposed to drive the PLCγ molecule from a closed inactive to an open active state.

In a variety of eukaryotic cells, Src kinases are responsible for the Y783 phosphorylation event [24], [25], [26], and a direct association between a sea urchin Src family kinase (SFK1) and PLCγ has been detected *in vitro* [27]. Src kinases are themselves regulated by phosphorylation; they have an inhibitory site at Y527 and an auto-activation site at Y416 [28]. The C-terminal Y527 site is phosphorylated by Csk [29], rendering the kinase in an auto-inhibited conformation. Dephosphorylation of Y527 followed by the auto-phosphorylation of Y416 leads to Src activation. Thus, though Src kinases are not traditionally thought to be regulators of membrane fusion, they could nonetheless provide a mechanistic link between GTP hydrolysis and PLCγ activation during NE assembly [30]. Therefore, we hypothesised that during NE formation, an SFK is responsible for phosphorylating PLCγ on its Y783 residue to achieve full PLCγ catalytic activity [21]. To test this we used time resolved Förster resonance energy transfer (FRET) measured by single and two-photon fluorescence lifetime imaging microscopy (FLIM) to examine the proposed SFK-PLCγ interaction *in vivo* and *in vitro*, and pharmacological studies to assess the SFK-dependency of NE assembly.

Our findings show the direct interaction and temporal regulation of PLCγ and SFK1 *in vivo* by time-resolved FRET. We also demonstrate that as a prerequisite for protein activation, there is a rapid phosphorylation of PLCγ on its Y783 residue in response to GTP *in vitro.* The ensemble of our data show the phosphorylation and activation of PLCγ by SFK1 during NE assembly both *in vivo* and *in vitro*.

**Figure 3:**
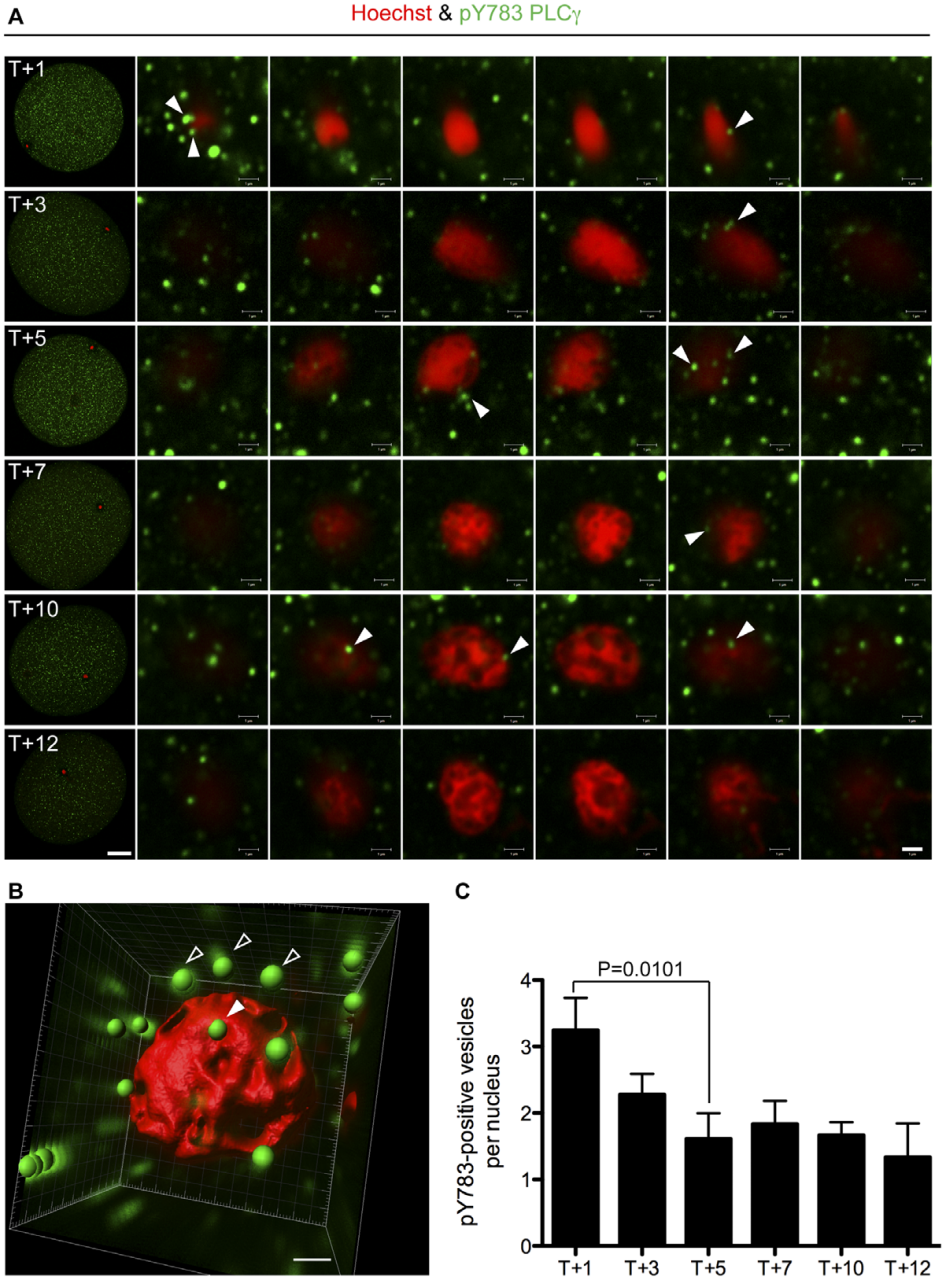
PLCγ is transiently phosphorylated on Y783 at the NE *in vivo*. (**A**) Fertilised (T1–12) *L. pictus* eggs were fixed and stained with anti-pY783 on PLCγ, (green) and Hoechst (red), and imaged by confocal microscopy. Arrows (white) indicate pY783 positive vesicles recruited to the forming MPN. Data are representative of those obtained in three independent experiments. Scale bar is 20 µm (whole egg) or 1 µm (20× zoom). (**B**) Z-series from (A) were manipulated in Imaris (see Methods) to form a 3D reconstruction. Membrane vesicles in contact with the nucleus surface were scored (solid arrow). Those in close proximity were not (open arrow). (**C**) Quantification of the data in A, B. Vesicles were scored in three independent experiments. Data are expressed as mean+s.e.m.

**Figure 4:**
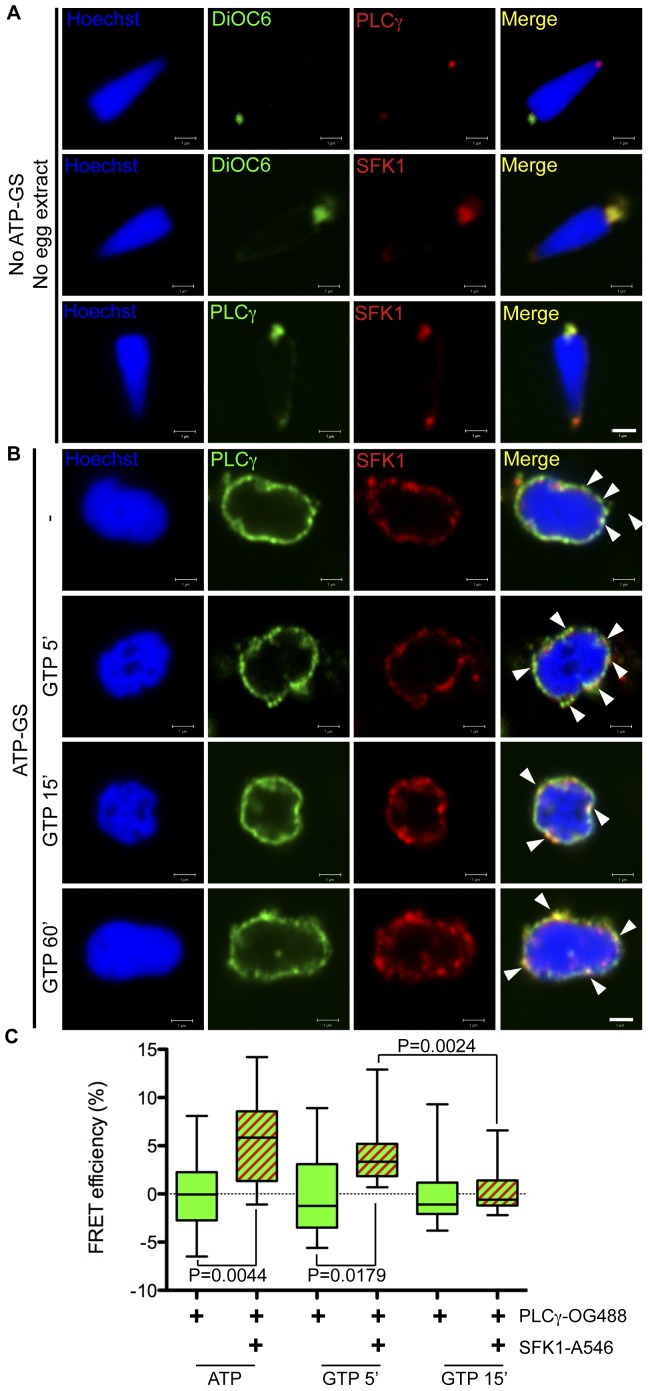
Interaction of PLCγ and SFK1 on the MPN surface *in* *vitro* declines after GTP addition. (**A**) Condensed demembranated sperm nuclei were fixed and labelled with anti-PLCγ or anti-SFK1 (red), Hoechst (blue) and DiOC_6_ (green). Nuclei were imaged by confocal microscopy. (**B**) Demembranated sperm nuclei were decondensed in fertilised egg cytoplasmic extract and ATP-GS. Nuclei were treated with 1 mM GTP as indicated (minutes), fixed and labelled with anti-PLCγ (green) and anti-SFK1 (red) directly conjugated to OG488 and Cy5 respectively. Arrows denote PLCγ and SFK1 co-localisation. All images are representative of those obtained in three independent experiments. Scale bar is 1 µm. (**C**) Decondensed nuclei were prepared as in B, and labelled with anti-PLCγ and anti-SFK directly conjugated to OG488 (green, donor) and Alexa 546 (red, acceptor) respectively. The τ_p_ and τ_m_ of the donor chromophore were determined for each condition by frequency domain FLIM in the absence (green) and presence (green and red bars) of acceptor and used to calculate the FRET efficiency. 10–18 nuclei were analysed for each condition. Boxes display the median, upper and lower quartiles and whiskers the maximum and minimum values.

**Figure 5:**
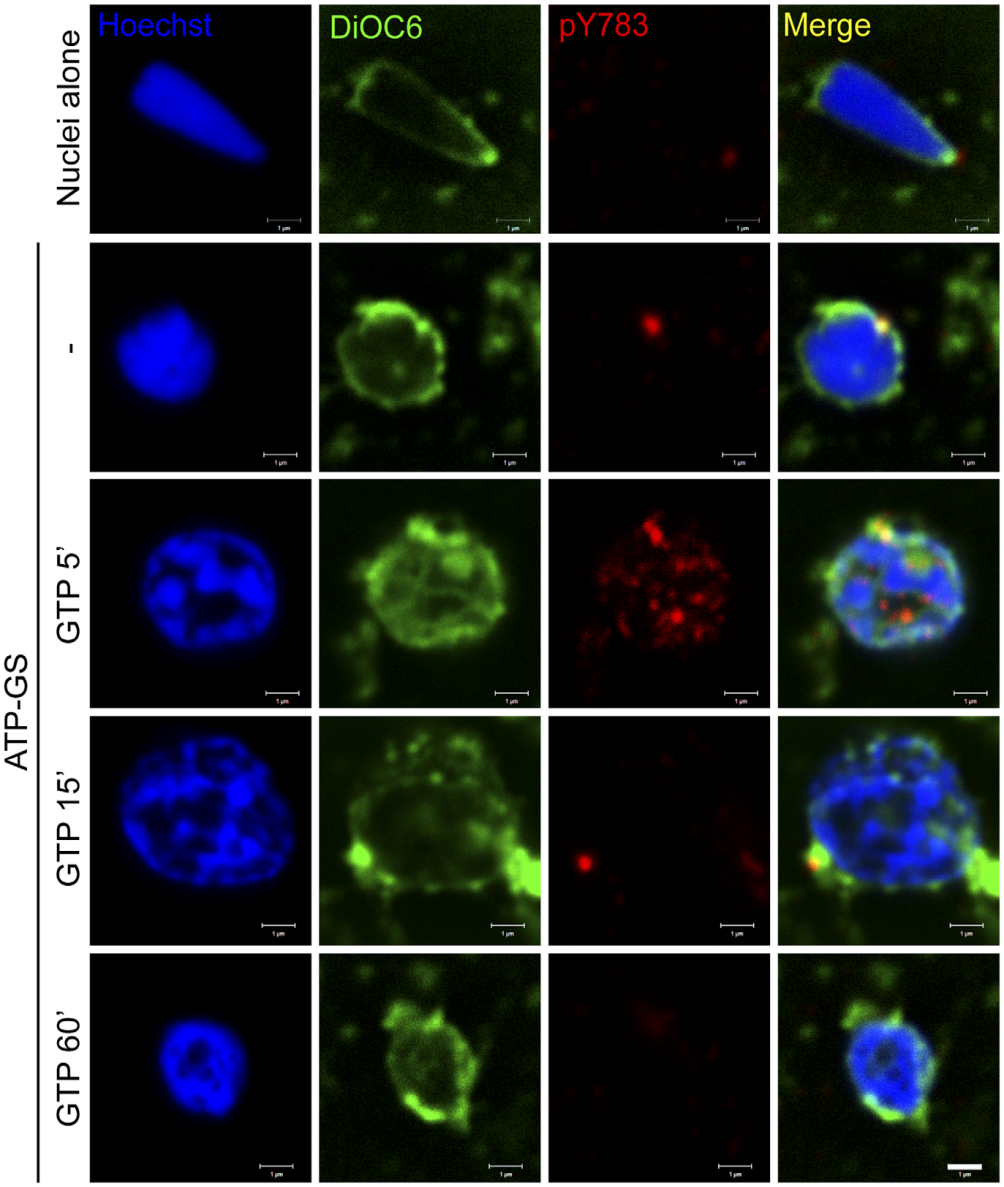
PLCγ is transiently phosphorylated on Y783 at the NE *in vitro*. Demembranated sperm nuclei were fixed alone (top row) or decondensed in fertilised egg cytoplasmic extract and ATP-GS (lower panels). Nuclei were additionally treated with 1 mM GTP for the times indicated (minutes) and were fixed and stained with Hoechst (blue), DiOC_6_ (green) and anti-pY783 of PLCγ (red). Nuclei were imaged by confocal microscopy. Note the staining of the NERs with anti-pY783 is retained in nuclei decondensed in ATP-GS (2^nd^ row). Data are representative of those obtained in three independent experiments. Scale bar is 1 µm.

## Materials and Methods

### Sea Urchin Gametes, Demembranated Sperm Nuclei and Egg Extract Preparation

*Lytechinus pictus* were purchased from Marinus Scientific (Long Beach, CA). Gamete shedding and collection took place as described [31], [32]. Fertilised egg cytoplasmic (S10) extracts and demembranated sperm nuclei (0.1% Triton X-100 extracted) were prepared as previously described [31].

**Figure 6:**
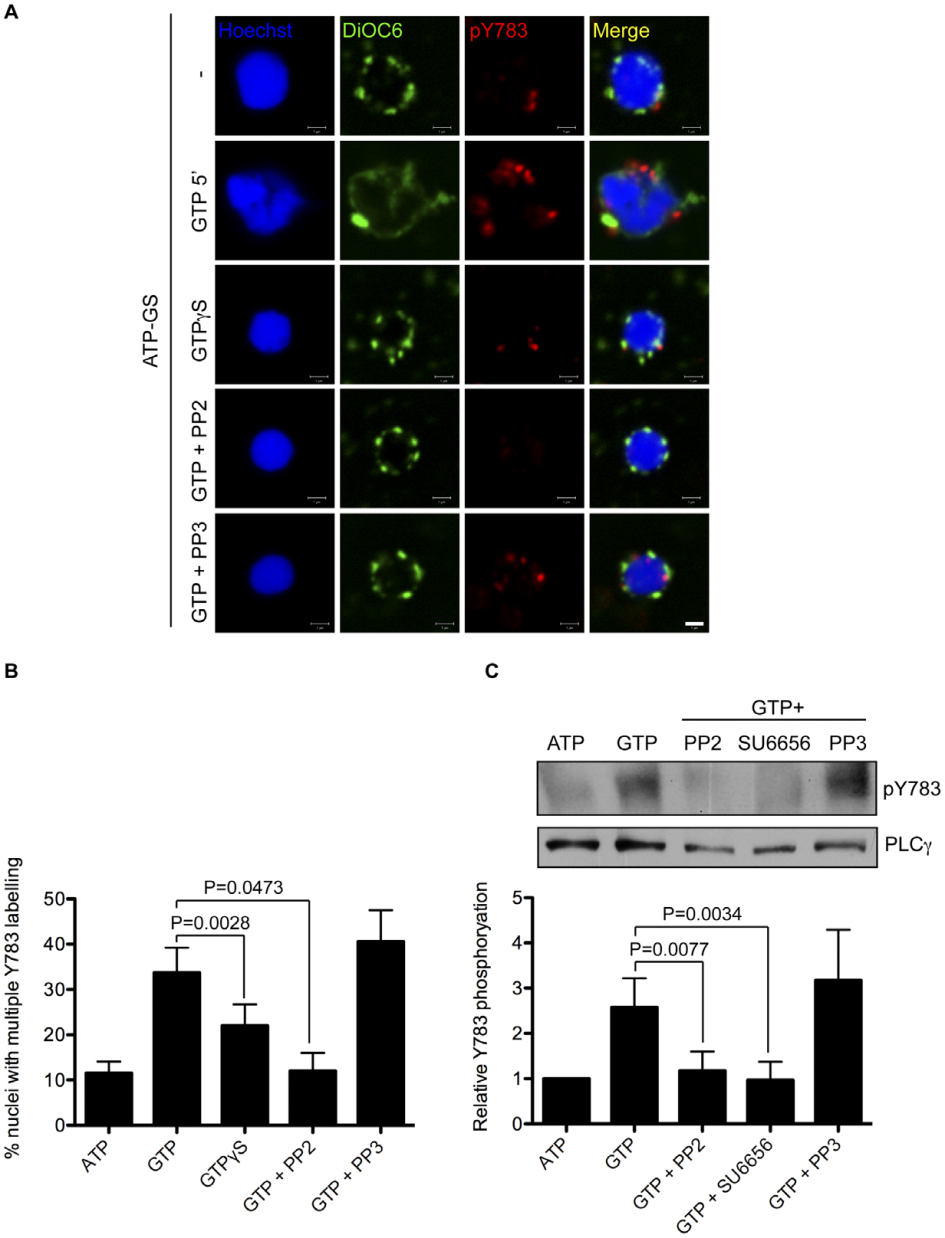
PLCγ Y783 phosphorylation requires SFK catalytic activity and GTP hydrolysis. (**A**) Experiment was performed as in Figure. 5 with nuclei treated for 5 minutes with 1 mM GTP or 2 mM GTPγS. Nuclei were also pre-incubated with the Src inhibitor PP2 or its inactive analogue PP3 (both 10 µM). After this time nuclei were fixed and stained with Hoechst (blue), DiOC_6_ (green) and anti-pY783 (red), and imaged by confocal microscopy. (**B**) Nuclei in A were scored for multiple pY783-positive sites on the nucleus surface. Data are expressed as mean+s.e.m from three independent experiments. Scale bar is 1 µm. (**C**) Experiments performed as in (B) with NE proteins subjected to an anti-pY783 western blot. pY783 bands were normalised to total PLCγ and expressed as a fold-change compared to nuclei assembled in the presence of ATP only. Data are expressed as mean+s.e.m from at least three independent experiments.

**Figure 7:**
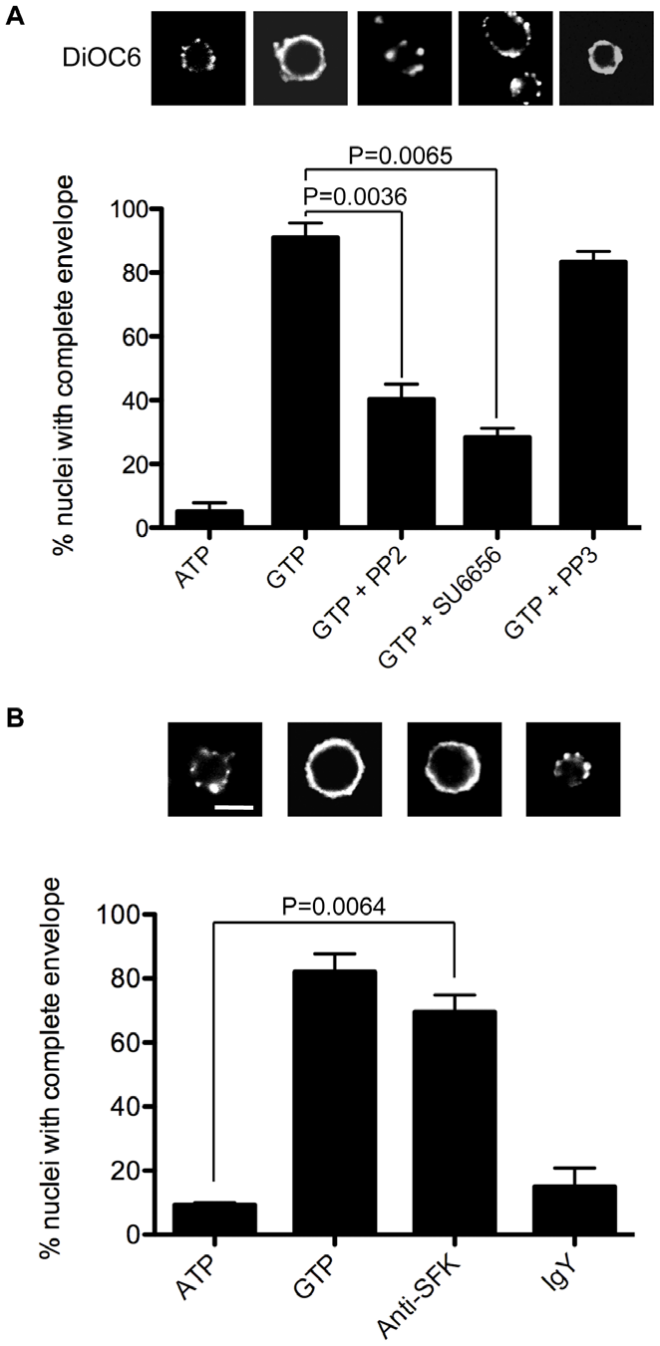
NE formation *in vitro* is dependent upon SFK kinase activity. (**A**) Experiments were performed as in Figure 6 with incubation of 1 mM GTP for 2 hours. After this time nuclear membranes were stained with DiOC_6_, and the nuclei scored as having bound vesicles (punctate signal) or a complete NE (continuous rim). Epifluoresence images and quantification (mean+s.e.m) are for three independent experiments. (**B**) Experiment performed as in A with GTP replaced with either normal IgY or anti-SFK1 serum (both at 1 µg/ml). Nuclei were scored as above. Scale bar is 5 µm.

**Figure 8:**
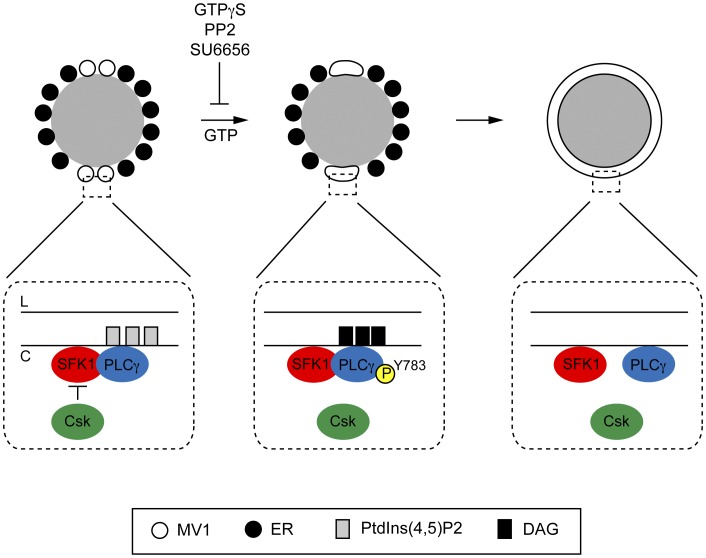
Schematic model and data summary of molecular events leading to NE formation. Once the sperm has entered the egg in vivo, or upon the sperm nucleus mixing with egg cytoplasmic extract and ATP-GS in vitro, the sperm nucleus decondenses (not shown), with concomitant membrane vesicle binding to the nucleus surface (grey). **Left**: MV1 vesicles enriched in SFK1, PLCγ and PtdIns(4,5)P_2_ bind in a polar fashion near the two sperm NERs. SFK1 and PLCγ directly interact. The premature phosphorylation of PLCγ by SFK1 is prevented by Csk inhibition of SFK1. **Middle**: Upon addition of GTP, Csk inhibition of SFK1 is removed, and SFK1 phosphorylates PLCγ on its Y783 residue, a pre-requisite for full PLCγ activation. Once active, PLCγ hydrolyses PtdIns(4,5)P_2_ to the fusogenic lipid DAG, which initially promotes membrane fusion events between MV1 vesicles and possibly NERs. **Right**: Fusion events subsequently spread over the surface of the sperm nucleus [19] until it is enclosed by a continuous double bilayer perforated with nuclear pore complexes (not shown). After fusion is initiated, PLCγ and SFK1 dissociate but remain on the NE. L and C denote the luminal and cytoplasmic faces of vesicles respectively. ER denotes endoplasmic reticulum derived membranes.

### Sea Urchin Egg Time Series

Eggs were collected in Millipore filtered sea water (MPSW) and dejellied by passing twice through a 210 µm Nytex mesh [32]. Eggs were fertilised and grown on a rotary shaker at 20°C. At the required time an aliquot of egg suspension was removed, mixed with 1 volume of 7.4% paraformaldehyde (PFA) in ice-cold isolation buffer (80 mM PIPES, pH 7.2, 5 mM EGTA, 5 mM MgCl_2_, 1 M glycerol), fixed for 1 hour at 20°C with shaking, centrifuged at 150 g, 4°C for 1 minute and the resulting egg pellet washed twice in PBS. Eggs were finally resuspended in 0.05% azide in PBS and stored at 4°C.

### Cell-free Assembly of the Male Pronuclear Envelope

20 µl of S10 cytoplasmic extract was combined with 1×10^6^ demembranated sperm nuclei and 1.2 µl of ATP generating system (ATP-GS) for 1 hour at room temperature after which nucleus decondensation was confirmed by light microscopy. The addition of 1 mM GTP (final concentration) triggered the fusion of membrane vesicles bound to the surface of nuclei to form a double bilayer.

For analysis of the NE by confocal microscopy, reactions were terminated by diluting samples 7-fold in ice-cold lysis buffer (10 mM Hepes pH 8.0, 250 mM NaCl, 5 mM MgCl_2_, 110 mM glycine, 250 mM glycerol, 1 mM DTT, 1 mM AEBSF). Nuclei were settled onto poly-L-lysine coated coverslips (BD Biosciences) and fixed for 10 minutes in 3.7% PFA in TN buffer (10 mM Tris-HCl, pH 7.2, 150 mM NaCl). Samples were blocked in 3% fatty acid free BSA (Sigma) in PBS for 15 minutes. The sperm nucleus and associated membranes were labelled with Hoechst 33342 (2 µg/ml) and 2 µM DiOC_6_ (Invitrogen) respectively for 10 minutes each in PBS. Proteins were labelled by incubation with antibodies against PLCγ (1∶500), SFK1 (1∶750) or pY783 (1∶50, Santa Cruz). The anti-PLCγ and anti-SFK1 affinity-purified antibodies were kindly provided by Foltz, UCSB, and have been previously described [33], [34]. Visualisation of primary antibodies was by conjugation to DyLight 488 or 649 (Stratech). Secondary antibodies alone incubated with either nuclei or eggs did not produce a signal (Figure S1). If PLCγ and SFK1 were imaged together, the antibodies were directly conjugated to Oregon Green 488 (Invitrogen) and Cy5 (GE Healthcare) respectively. Slides were mounted in ProLong Gold reagent (Invitrogen). Images were acquired on a Zeiss 710 upright confocal microscope run by Zen software (2009), with a 63×/1.4NA oil-immersion objective and the custom filter set-up for the probes indicated. Chromophore and filter set compatibility was tested to ensure direct excitation or bleed-through did not occur. In experiments where nuclei were scored for a particular characteristic, 20–54 nuclei were scored in each of 3 independent experiments.

To assess the effects of pharmacological inhibitors and antibodies on NE assembly, nuclei were labelled with DiOC_6_ and examined by epifluorescence microscopy. This end-point assay allows a rapid and efficient screening of large numbers of agents for their effect of NE formation and many nuclei can be assessed for each data point, thus making for a robust assay when combined with appropriate statistical analysis.

For western analyses, reactions were scaled up 5-fold in volume. At the time indicated, samples were underlaid with 1 volume of 0.5 M sucrose/TN solution and centrifuged at 750 g, 4°C for 15 minutes. The pellet was resuspended in 75 µl LB/0.1% Triton X-100/complete mini protease inhibitors (Roche) and incubated on ice for 15 minutes. Samples were centrifuged at 5000 g, 4°C for 5 minutes and the supernatant containing NE proteins removed, mixed with 25 µl 4×SDS buffer and boiled for 10 minutes at 95°C. Samples were resolved on 4–12% bis-tris gels (Invitrogen), transferred to PVDF membranes and blotted for either PLCγ (1∶1000, Millipore), pY783 (1∶500), or SFK1 pY527 (1∶1000, NEB). Protein was visualised with ECL. Band densitometry from 3 independent experiments took place with ImageJ v1.42.

In experiments where pY783 status was manipulated, PP2, PP3 and SU6656 (all 10 µM) were added 15 minutes prior to GTP. GTPγS (2 mM) was applied in place of GTP. While PP2 and SU6656 are not specific to Src kinases, they both inhibit Src preferentially (IC_50_ values are >5-fold lower than for non-Src targets).

### Confocal Microscopy of Male Pronuclear Envelope Formation *in vivo*

All steps took place at room temperature. Fixed eggs were settled onto freshly prepared 0.005% poly-L-lysine coverslips for 15 minutes, and blocked/permeabilised in 3% fatty acid free BSA/0.5% saponin in PBS for 15 minutes. Eggs were quenched in 1 mg/ml sodium borohydride for 2 minutes. Slides were labelled with anti-PLCγ or anti-pY783 and visualised with DyLight 488, and anti-SFK1 directly conjugated to Cy5. Antibody incubations were for 1 hour in 3% fatty acid free BSA/0.5% saponin in PBS. Eggs were additionally labelled with Hoechst 33342 and mounted in ProLong Gold. Z-series spanning the entire nucleus were acquired on a Zeiss 710 upright confocal microscope with a 63×/1.4 NA oil-immersion objective as described above. Optical sections were 0.8 µm thick and taken 0.8 µm apart. For the scoring of pY783 positive vesicles on the sperm nucleus surface, images were manipulated in Imaris (v 7.3.1). Briefly, the Hoechst channel was used to create an artificial ‘surface’ over the nucleus, while the pY783 signal was labelled as ‘spots’ of ∼0.5 µm diameter, the size of NE precursor membrane vesicles as previously reported [12]. Only labelled vesicles that were in contact with the surface of the nucleus were scored. This process was repeated in nine eggs from three independently prepared time series.

### Conjugation of Oregon Green 488, Alexa 546 and Cy5 to Antibodies

100 µg of antibody was conjugated to 25 µg of chromophore in 100 µl adjusted to pH 8.5 with 50 mM sodium borate buffer. Oregon Green 488 (OG488, donor chromophore) was conjugated to anti-PLCγ, and anti-SFK1 was conjugated to either Alexa 546, Alexa 594 or Cy5 (acceptor) for 1 hour in the dark at room temperature. Free chromophore was separated from antibody-dye conjugate with a Nanosep column (Merck Biosciences) containing 50 µl separation resin (Pierce). The dye:protein ratio of the chromophore-antibody conjugate was calculated as per manufacturer’s instructions. Conjugates had a dye:protein ratio of <4∶1. All conjugated antibodies were tested to ensure antigen recognition was unaffected by the presence of chromophore (Figure S2).

### Time Resolved FRET Measured by Two-photon FLIM

Fixed eggs were settled, blocked, permeabilised and quenched as described above. Protein labelling took place with anti-PLCγ-OG488 and anti-SFK1-Cy5 conjugates. Samples were labelled in 3% fatty acid free BSA/0.5% saponin in PBS for 2 hours. Eggs were additionally labelled with Hoechst 33342 and mounted in ProLong Gold. As samples were fixed prior to labelling with chromophores, fixation cannot create false-positives in this assay by ‘bringing together’ chromophores. Moreover, our work has demonstrated that fixation has no effect on protein-protein FRET compared to live studies [35], [36]. Images were acquired on a Nikon TE2000-E inverted microscope with a 60×/1.49NA oil immersion objective and a Hamamatsu ORCA camera. Samples were excited with the 920 nm laser line of a 6 W Mira (Coherent) laser at a power of 27 mW, and were subjected to a maximum of 1600 scans to obtain a sufficient number of photons (>100) for a bi-exponential Marquardt curve fit. The scanning number was controlled to ensure no photobleaching occurred. Only in two-photon excitation these donor and acceptor pairs are ideal as the second emission spectrum of OG488 overlaps adequately with the excitation spectrum of Cy5 and the 920 nm excitation does not directly excite Cy5. Calculation of the donor chromophore (OG488) lifetime was performed in TRI2 custom software (Paul Barber, Dept. of Oncology, University of Oxford). Here, the presence of FRET between donor and acceptor chromophore is expressed as FRET efficiency (*E*; Eqn. 1), calculated from the donor lifetime (τ) obtained in each experimental condition [donor alone (D) and donor plus acceptor (DA)] as:
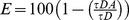
(1)


The anti-SFK1 antibody used in this study may also recognise SFK7 as both have similar C-terminal amino acid sequences. However, Townley *et al.*, were only able to detect *in vitro* a direct interaction between PLCγ and SFK1, not SFK7 [27]. Thus in the FRET assays the only relevant interaction measured will be between PLCγ and SFK1.

### FRET Measured by Single-photon Multi-frequency Domain FLIM

Nuclei were decondensed in S10 with ATP-GS as described above and samples treated with 1 mM GTP. Reactions were terminated, and nuclei settled onto coverslips, fixed, blocked and quenched as described above. Samples were labelled with anti-PLCγ-OG488 alone or additionally with anti-SFK1-Alexa 546. Nuclei were stained with Hoechst 33342 and mounted in ProLong Gold.

Nuclei were located with the mercury (Hg) source using a DAPI filter set and the images acquired on a Nikon Eclipse Ti inverted microscope with a 60×/1.49NA oil immersion objective together with a LI^2^-CAM camera (Lambert Instruments). Anti-PLCγ-OG488 labelled samples were excited with a sinusoidally modulated 473 nm line of an Omicron blue diode laser (Photon Lines) modulated at 40 MHz at an optimum angle of 140°. Between 10 and 18 nuclei were analysed for each condition. For each nucleus the τ phase (τ_p_) and τ modulation (τ_m_) values were calculated in LI-FLIM v1.2.9 software (Lambert Instruments). In these experiments τ_p_ and τ_m_ values were ≤0.20 ns apart, so the values were averaged (Eqn. 2) for the FRET efficiency calculation:
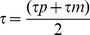
(2)


### Statistical Analysis

Data was analysed in Graphpad Prism v5.0c. Datasets were subjected to unpaired 2-tailed t-tests (FRET experiments) or paired 1- or 2-tailed t-tests with 95% confidence intervals.

## Results

### PLCγ and SFK1 Co-localise on Membrane Vesicles in the Egg Cortex and at the Surface of the MPN *in vivo*

To test whether the PLCγ/SFK1 enriched vesicles participate in NE assembly *in vivo*, eggs were fertilised and fixed at various times up to 15 minutes post- fertilisation (at which time male and female pronuclear fusion occurs). Fixed eggs were labelled with PLCγ and SFK1 antibodies and imaged by confocal microscopy. The data in Figure 1 show that in unfertilised eggs (T0) PLCγ and SFK1 co-localise on punctate structures in the egg cortex. After fertilisation both PLCγ and SFK1 appear in the vicinity of the decondensing sperm nucleus on the same vesicle population (white arrows). At early time points (T+1 to T+7) the vesicles adjacent to the MPN surface appeared to be those labelled most intensely for PLCγ and SFK1. At later points (T+10 to T+15) while co-labelled vesicles were still observed on the sperm nucleus surface, they appeared not to be brighter than the other vesicles in the field of view. An example of a nucleus showing all optical sections is shown in Figure S3.

Although the proteins were co-localised on the nuclear surface, confocal microscopy does not provide the means to detect an interaction between PLCγ and SFK1. To detect an association between PLCγ and SFK1 we used time-resolved FRET measured by two-photon FLIM. By this method, the interaction between PLCγ and SFK1 (<10 nm apart) would result in a transfer of energy from the donor chromophore (Oregon green 488 [OG488] coupled to anti-PLCγ) to the acceptor (Cy5 coupled to anti-SFK1). This energy transfer results in a decrease in the donor chromophore lifetime, and an increase in its FRET efficiency (see Methods for calculation). In the unfertilised egg (T0) we detected a significant increase in the donor FRET efficiency in the presence of the acceptor, indicative of FRET (Table 1, Fig. 2A top panels, B). Thus, in the unfertilised egg PLCγ and SFK1 interact. Moreover, the egg ‘heat-map’ indicated that the main region of interaction of the two proteins was the egg cortex, as this is where the greatest number of pixels with a short donor lifetime (<2.4 ns, pseudo-coloured red) was observed (Figure 2A). Specificity was demonstrated by lack of FRET between OG488-labelled anti-PLCγ and Cy5- labelled normal IgY; the latter binds non-specifically. In a repetition of these experiments on eggs three minutes post-fertilisation (T+3) FRET was detected in both the egg cytoplasm and the region around the MPN (Table 1). Thus PLCγ and SFK1 were interacting at this time point. At five minutes (T+5) post-fertilisation FRET was not detected between the chromophore labelled PLCγ and SFK1, in either the whole egg cytoplasm (Figure 2A, middle panels) or more specifically the region around the MPN (Figure 2A, lower panels) as indicated by lack of significant change in the calculated FRET efficiencies (Figure 2B, Table 1). Together the confocal and FRET analyses indicate that at T+5, PLCγ and SFK1, though still co-localised are not interacting.

### Y783 Undergoes Reversible Phosphorylation at the NE during Fertilisation *in vivo*

The temporally regulated direct association of PLCγ and SFK1 indicated that SFK1 may be responsible for the phosphorylation of PLCγ on residue Y783. Thus we predicted Y783 phosphorylation might show similar kinetics. To examine this we detected and quantified by a combination of confocal microscopy and image analysis the pY783 signal on the sperm nucleus during MPN formation. Figure 3A shows vesicles containing the pY783 signal detected by confocal microscopy at various time points post-fertilisation on the MPN surface. The acquired z-series was used to generate a 3D reconstruction of the sperm nucleus surface, and vesicles in contact with the nucleus quantified (Figure 3B). The total number of pY783 positive vesicles detected per nucleus decreased significantly during MPN formation by T+5 (Figure 3C). Thus during MPN assembly *in vivo*, PLCγ was retained on the nucleus surface, but the number of pY783 positive vesicles was diminished.

### PLCγ and SFK1 are Co-recruited to the NE in a Cell-free Assay

Given the correlation between Y783 phosphorylation kinetics, and the SFK1/PLCγ association, we tested this tyrosine phosphorylation event for susceptibility to SFK inhibitors. However, this was not possible *in vivo* because SFK is required for many other cellular events [27], [33], [37], [38], [39]. Therefore we tested the role of SFK1 in PLCγ Y783 phosphorylation in our cell-free assay. As the demembranated sperm nuclei in this assay bind NE precursor membrane vesicles specifically [6], [40], [41], this assay offered the advantage of allowing SFK-dependent events critical for NE assembly to probed in isolation. Moreover, as MPN formation proceeds similarly *in vivo* and *in vitro*, findings from the cell-free assay are applicable to NE assembly *in vivo* [11], [42].

Initially we examined whether PLCγ and SFK1 could be detected together on the sperm nucleus *in vitro*. Mixing of demembranated sperm nuclei with an active egg cytoplasmic extract (containing soluble proteins and NE precursor membrane vesicles) in the presence of an ATP generating system (ATP-GS) leads to the decondensation of the sperm nucleus with simultaneous binding of NE precursor membrane vesicles. Nuclei were subsequently imaged by confocal microscopy for the presence of PLCγ and SFK1. Total membrane was stained with the fluorescent lipophilic dye DiOC_6_. The data presented in Figure 4A show PLCγ and SFK1 were present on the NERs (the remnants of the sperm nuclear envelope) of demembranated nuclei. However, there was a marked recruitment of PLCγ and SFK1 over the entire surface of nuclei when they were incubated in the egg extract (Figure 4B). On the nuclear surface several points of co-localisation between PLCγ and SFK1 were observed. Upon the addition of GTP to induce fusion of the bound membranes, the localisation of PLCγ and SFK1 remained the same and a co-localisation of both proteins was observed as long as 60 minutes post-GTP addition. Based on data from our FRET-based fusion assay, by this time a complete NE has been formed [19].

### Interaction of PLCγ and SFK1 *in vitro* is Temporally Regulated

While the localisation of PLCγ and SFK1 appeared to be unchanged during NE formation, it was possible that as *in vivo*, the two proteins were undergoing a reversible interaction not detectable by confocal microscopy. To test this we examined the putative interaction of PLCγ and SFK1 in the cell-free assay by FRET detected by multiple-frequency domain FLIM. NE precursor membranes were bound to nuclei and PLCγ and SFK1 were labelled with antibodies conjugated to the chromophores OG488 (donor) and Alexa 546 (acceptor) respectively (Figure S4). The results in Figure 4C show that on vesicles bound to the nucleus surface (ATP), FRET was detected between PLCγ and SFK1-bound chromophore-conjugated antibodies, as indicated by a significant increase in the FRET efficiency (mean change from 0 to 5.7% in donor alone and donor plus acceptor conditions respectively). Five minutes after the addition of GTP a significant FRET efficiency was still evident. However 15 minutes after addition of GTP, FRET was not observed. Thus between 5 and 15 minutes after the addition of GTP to the cell-free assay, PLCγ and SFK1 dissociate but remain at the NE.

Thus far PLCγ and SFK1 behave in a similar manner *in vivo* and *in vitro*. To examine if Y783 also underwent a reversible phosphorylation event *in vitro* we examined the various stages of the assembly process by probing sperm nuclei for pY783 (Figure 5). First, we observed a pY783 signal in NERs of condensed demembranated nuclei, and nuclei to which NE precursor membrane vesicles had bound (ATP-GS) but not fused. The latter probably reflects pY783 of the NERs which may be important for NER incorporation by fusion into the forming male pronuclear envelope [7], [16]. After the addition of GTP we observed a striking increase in pY783 signal around the nuclear periphery by 5 minutes, which returned to its original level or lower by 15 minutes or later.

### PLCγ Y783 Phosphorylation is Dependent Upon SFK Catalytic Activity and GTP Hydrolysis

Having demonstrated the interaction of SFK1 with PLCγ, and that the reversible phosphorylation of Y783 on PLCγ was taking place during male pronuclear envelope assembly *in vivo* and *in vitro*, we hypothesised that Y783 phosphorylation was under the control of SFK1. To test this hypothesis, the phosphorylation of Y783 was triggered with GTP for 5 minutes (Figure 6A, B). To demonstrate that SFK1 was phosphorylating PLCγ, extracts were pre-incubated with the SFK inhibitor PP2 (or its inactive analogue PP3) prior to GTP addition. As predicted, PP2 abolished Y783 phosphorylation, while its inactive analogue did not, indicating SFK kinase activity was required for Y783 phosphorylation. To establish that GTP hydrolysis was necessary for pY783 phosphorylation, we replaced GTP with its slowly hydrolysing analogue GTPγS, an inhibitor of NE assembly. GTPγS was unable to adequately substitute for GTP, and in its presence significantly fewer nuclei displayed pY783 phosphorylation compared to GTP (22% compared to 34%).

These data were corroborated by western blot analysis of Y783 status on NEs (Figure 6C). GTP was shown to induce a 2.5-fold rise in Y783 phosphorylation that was susceptible to inhibition by PP2 and a different SFK inhibitor, SU6656 (but not PP3). Both inhibitors totally abolished GTP-induced Y783 phosphorylation. These data together confirm that SFK catalytic activity on male pronuclei is required for the phosphorylation of PLCγ on Y783.

### SFK Inhibition Blocks NE Formation

We previously demonstrated that the activation of PLCγ and hydrolysis of PtdIns(4,5)P_2_ to the fusogenic lipid DAG is a critical step in male pronuclear envelope formation [18], [19], [20], [43]. Therefore, if SFK activity were vital for PLCγ phosphorylation and activation, it would also be required for NE assembly. To test this, nuclei were scored for complete NEs after the addition of GTP in the presence and absence of SFK inhibitors (see Methods). GTP induced a complete NE in almost all (90%) nuclei observed (Figure 7A). NE assembly was significantly inhibited by both PP2 (40%) and SU6656 (<30%), but not PP3 (>80%).

Src kinases have a conserved inhibitory tyrosine residue at their C-terminus, Y527 [44]. When Y527 is phosphorylated, the kinase is inactive; Y527 dephosphorylation promotes kinase activation by removing its auto-inhibition. The anti-SFK1 antigenic sequence spans this inhibitory site, and this antibody has previously been shown to activate SFK1 in *Lytechinus variegatus* eggs and egg extracts [33]. Therefore, we predicted the antibody would mask the site, sterically hindering access to the Y527 kinase. NEs were therefore assembled around demembranated sperm nuclei and the ability of anti-SFK1 to induce NE formation was compared to the classical GTP trigger (Figure 7B). GTP induced NE formation in almost all nuclei (>80%). The affinity purified anti-SFK1 antibody was similarly effective (in the absence of GTP), with approximately 70% of nuclei displaying a complete envelope. As predicted, this envelope formation was accompanied by a reduction in the phosphorylation of the SFK1 Tyr527 site (Figure S5). The effect was specific to the antibody, as normal IgY did not have a significant effect (P = 0.3901) on envelope formation, and proteins other than the heavy and light chains were not observed in the anti-SFK1 antibody (Figure S5).

## Discussion

Two models have been proposed for how a NE forms. The ‘envelopment’ model [45] proposes ER envelops the nucleus, with remaining gaps closed by membranes coalescing around nuclear pore complexes [46]. This model does not account for membrane fusion events that may be required to seal gaps left in the double bilayer. The second is the ‘fusion’ model [14], [15], [47]. As in the envelopment model, the ER provides the bulk of the membrane needed to form the NE. However, a second membrane source in the form of vesicles is indispensible for the formation of the NE, and membrane fusion originates from this fraction [19]. The fraction, termed MV1 in the sea urchin, is enriched in PLCγ and its substrate PtdIns(4,5)P_2_ [12]. In the fusion model, PLCγ, activated in response to GTP hydrolysis, subsequently hydrolyses PtdIns(4,5)P_2_ to the fusogenic lipid DAG. However, the steps leading to PLCγ activation have remained unclear. In this study we have taken a multi-disciplinary approach to address this issue, and as a result we propose a refined model of the timing of early molecular events during NE formation (Figure 8).

We observed a co-localisation of PLCγ with SFK1 on vesicles in the cortex of unfertilised sea urchin eggs. We suggest that these vesicles are MV1, which as isolated are highly enriched in both PLCγ and SFK1 [12], [48]. FRET experiments demonstrate that PLCγ and SFK1 were interacting on these vesicles. Controls employing normal IgY demonstrated the assay for PLCγ-SFK1 interaction is specific. Some of these vesicles were present in the vicinity of the male pronucleus *in vivo.* The recruitment of PLCγ and SFK1 to the MPN was clearly shown in the cell-free assay (Figure 4A). PLCγ and SFK1 remain on the forming NE for at least 10 minutes after they cease associating (at T+5). At approximately 15 minutes post-fertilisation the male and female pronuclei undergo zygote fusion, an event also requiring membrane fusion [7]. It is possible that the retention of PLCγ and SFK1 in close proximity on the NE facilitates their re-association to generate a second round of fusogenic lipids for male-female pronuclear fusion.

The co-recruitment of PLCγ and SFK1 to the NE and their subsequent dissociation are very similar kinetically to the Y783 phosphorylation of PLCγ. This is highly consistent with our hypothesis that SFK1 is responsible for phosphorylating PLCγ on Y783, an event necessary for full PLCγ activity [21]. SFK inhibitors blocked Y783 phosphorylation and NE assembly *in vitro*, confirming this hypothesis.

It should be noted that PLCγ-SFK1 association-dissociation, and Y783 phosphorylation kinetics differ *in vivo* versus *in vitro*. This is likely to result from MPN formation proceeding more slowly *in vitro* than *in vivo* (60 minutes vs <15 minutes respectively) perhaps because cell-free extracts lack the cytoskeletal co-ordination present *in vivo* or because of the dilution of cytoplasm occurring during extract preparation. Thus, rather than vesicles being specifically ‘trafficked’ to the nuclear surface they would have to bind after diffusion/collision. Nonetheless, the ‘peak’ pY783 signal systematically follows the PLCγ-SFK1 association whether *in vitro* or *in vivo*.

A further observation on Y783 phosphorylation *in vivo* is that after PLCγ and SFK1 dissociate there is a specific drop in the pY783 signal at the reforming NE whereas the global pY783 signal persisted in the egg. It is likely that this is a reflection of different pools of active PLCγ in the egg shortly after fertilisation, and while the pool at the NE diminishes, others are maintained for other specific purposes. PLCγ activation is well characterised during fertilisation, playing a pivotal role in calcium signalling in the sea urchin [49]. The identity of the kinase(s) maintaining the other pool(s) of active PLCγ is unknown. Other SFKs could be responsible, but since interactions between PLCγ and SFKs other than SFK1 have not been demonstrated [27], it is likely that other tyrosine kinases may be responsible.

While PLCγ and SFK1 are co-recruited to the NE *in vitro* and directly associate there, PLCγ is not phosphorylated until the addition of GTP to the extracts. Thus, there must be an inhibitory component that prevents the premature activation of SFK1. Phosphorylation of Src at Y527 by Csk inhibits Src kinase activity [29]. Two Csk isoforms have been identified in the *Strongylocentrotus purpuratus* sea urchin genome [50]. This observation, together with our experiments in Figure 7B and S5 indicate that SFK1 is activated in part by removing the phospho-inhibition of pY527 by a Csk kinase.

We have defined further the molecular machinery underlying the membrane fusion events of male pronuclear assembly its timing. The proteins upstream of SFK1 and PLCγ are still to be identified, including the GTPase(s) activated in response to GTP. GTP hydrolysis by Ran is a well-established event during NE assembly [51], but to date no study has linked Ran to SFK1 or PLCγ activation. It is possible that another class of GTPase is active during NE formation. Monomeric Rab GTPases are ubiquitous in membrane trafficking [52], sea urchin cortical granule and other exocytoses and cell division [53], [54]. An alternative possibility is that a heterotrimeric GTPase acts upstream of SFK1 activation. c-Src activity is enhanced *in vitro* by Gαs and Gαi subunits [55], and the activation of Gαs and Gαq heterotrimeric GTPases is required for Ca^2+^ mobilisation through the SFK-PLCγ pathway in sea urchin eggs, but the effect is mediated by the Gβγ components [56].

We have now demonstrated a role for MV1 in NE formation both *in vitro* [6] and *in vivo*. Given its lipid composition, and localisation throughout the egg cortex (a site of frequent endo- and exocytotic events) we have previously suggested a universal role for MV1 as a general facilitator of membrane fusion by virtue of its atypical proteo-lipid composition [15], its PLCγ activity held in abeyance until a tyrosine phosphroylation event. Such a ‘signalling platform’ role for early endosomes in mammalian cells has been suggested [57]. Thus MV1 may be a conserved ‘fusion platform’ vesicle population from echinoderms to mammals. We propose that the molecular mechanism outlined in this paper (Figure 8) is conserved, and serves not just to drive membrane fusion events that underlie the formation of echinoderm male pronuclei, but may also be used for mitotic nuclear envelope formation.

## Supporting Information

Figure S1 Decondensed sperm nuclei (left) or T0 (unfertilised) eggs (right) were prepared as described in the methods section, and stained with DyLight 488 and DyLight 649 alone (both at 1∶500) in the absence of primary antibodies. Samples were additionally stained with Hoechst 33342 and imaged by confocal microscopy. Note the blue channel of the egg image has been deliberately enhanced to show the dimensions of the egg. Scale bar 1 µm (nuclei) and 20 µm (egg).
(TIFF)

Figure S2 **Left**: Anti-SFK1 blot undertaken with antibodies in the absence and presence of Cy5 respectively. The antibody concentration was equal in both blots. Relevant *M_r_* are indicated (×10^3^). The arrow indicates the antigen of predicted size for SFK1. **Right**: T0 (unfertilised) eggs were prepared as described in the methods section, and stained with anti-PLCg plus a DyLight 488 secondary, anti-PLCγ conjugated to Oregon green 488, anti-SFK1 plus a DyLight 649 secondary or anti-SFK1 conjugated to Cy5. Samples were imaged by confocal microscopy. Conjugated antibodies continued to recognise vesicles in the egg cortex. As the FRET assay is a 2-site assay any non-specific signal/egg autofluoresence will not lead to a non-specific signal in our assay. Scale bar is 5 µm.
(TIFF)

Figure S3 A T+5 egg fixed, and labelled with Hoechst 33342, anti-PLCg followed by a DyLight 488 secondary and anti-SFK1-Cy5 direct conjugate. The complete confocal z-series is shown, encompassing the sperm nucleus. Scale bar is 1 µm.
(TIF)

Figure S4 Demembranated sperm nuclei were decondensed in fertilised egg cytoplasmic extract in the presence of ATP-GS. Nuclei were fixed and labelled with Hoechst 33342 (blue), anti-PLCγ and anti-SFK1 directly conjugated to OG488 (green) and Alexa 546 (red) respectively. The images shown were obtained by confocal microscopy to confirm the recruitment of PLCγ and SFK1 to the NE could be detected with the reagents for the subsequent FRET experiments. The co-localisation of PLCγ and SFK1 further confirms the retention of the specificity of the conjugated antisera. Scale bar is 1 µm.
(TIFF)

Figure S5 **Left**: *in vitro* NE formation assays were performed as described in the methods section. Membrane fusion events were induced with 1 mM GTP or 1 µg/ml anti-SFK1 antibody for 5 minutes. Nuclei were separated from unbound vesicles by centrifugation through a sucrose cushion, and nuclei pellets resuspended in 4× SDS buffer. The Tyr527 site of SFK1 was probed by western analysis with an anti-pTyr527 antibody, and band intensity quantified in Image J. **Right**: 3 µg anti-SFK1 was separated on a 4–12% pre-cast bis-tris gel and stained with colloidal coomassie blue stain (Thermo). The heavy (H) and light (L) chain of the affinity purified IgY were visualised. Note the IgY heavy chain is 70 kDa. *M_r_* is denoted in kDa.
(TIFF)

## References

[1] Poccia D, Collas P (1996). Transforming sperm nuclei into male pronuclei in vivo and in vitro.. Curr Top Dev Biol.

[2] Wilson EB (1925). The Sex-Chromosomes of Sea-Urchins.. Science.

[3] Poccia D, Collas P (1997). Nuclear envelope dynamics during male pronuclear development.. Dev Growth Differ.

[4] Longo FJ (1973). Fertilization: a comparative ultrastructural review.. Biol Reprod.

[5] Longo FJ (1981). Morphological features of the surface of the sea urchin (Arbacia punctulata) egg: oolemma-cortical granule association.. Dev Biol.

[6] Collas P, Poccia D (1996). Distinct egg membrane vesicles differing in binding and fusion properties contribute to sea urchin male pronuclear envelopes formed in vitro.. J Cell Sci.

[7] Longo FJ, Anderson E (1968). The fine structure of pronuclear development and fusion in the sea urchin, Arbacia punctulata.. J Cell Biol.

[8] Longo FJ (1976). Derivation of the membrane comprising the male pronuclear envelope in inseminated sea urchin eggs.. Dev Biol.

[9] Poccia DL, Green GR (1992). Packaging and unpackaging the sea urchin sperm genome.. Trends Biochem Sci.

[10] Imschenetzky M, Puchi M, Morin V, Medina R, Montecino M (2003). Chromatin remodeling during sea urchin early development: molecular determinants for pronuclei formation and transcriptional activation.. Gene.

[11] Cameron LA, Poccia DL (1994). In vitro development of the sea urchin male pronucleus.. Dev Biol.

[12] Byrne RD, Garnier-Lhomme M, Han K, Dowicki M, Michael N (2007). PLCgamma is enriched on poly-phosphoinositide-rich vesicles to control nuclear envelope assembly.. Cell Signal.

[13] Jaffe LA, Terasaki M (2004). Quantitative microinjection of oocytes, eggs, and embryos.. Methods Cell Biol.

[14] Byrne RD, Poccia DL, Larijani B (2009). Role of phospholipase C in nuclear envelope asembly.. Clin Lipidol.

[15] Larijani B, Poccia DL (2009). Nuclear envelope formation: mind the gaps.. Annu Rev Biophys.

[16] Collas P, Poccia D (1995). Lipophilic organizing structures of sperm nuclei target membrane vesicle binding and are incorporated into the nuclear envelope.. Dev Biol.

[17] Garnier-Lhomme M, Byrne RD, Hobday TM, Gschmeissner S, Woscholski R (2009). Nuclear envelope remnants: fluid membranes enriched in sterols and polyphosphoinositides.. PLoS One.

[18] Barona T, Byrne RD, Pettitt TR, Wakelam MJ, Larijani B (2005). Diacylglycerol induces fusion of nuclear envelope membrane precursor vesicles.. J Biol Chem.

[19] Dumas F, Byrne RD, Vincent B, Hobday TM, Poccia DL (2010). Spatial regulation of membrane fusion controlled by modification of phosphoinositides.. PLoS One.

[20] Larijani B, Barona TM, Poccia DL (2001). Role for phosphatidylinositol in nuclear envelope formation.. Biochem J.

[21] Kim HK, Kim JW, Zilberstein A, Margolis B, Kim JG (1991). PDGF stimulation of inositol phospholipid hydrolysis requires PLC-gamma 1 phosphorylation on tyrosine residues 783 and 1254.. Cell.

[22] Gresset A, Hicks SN, Harden TK, Sondek J (2010). Mechanism of phosphorylation-induced activation of phospholipase C-gamma isozymes.. J Biol Chem.

[23] Poulin B, Sekiya F, Rhee SG (2005). Intramolecular interaction between phosphorylated tyrosine-783 and the C-terminal Src homology 2 domain activates phospholipase C-gamma1.. Proc Natl Acad Sci U S A.

[24] Liao F, Shin HS, Rhee SG (1993). In vitro tyrosine phosphorylation of PLC-gamma 1 and PLC-gamma 2 by src-family protein tyrosine kinases.. Biochem Biophys Res Commun.

[25] Arkinstall S, Payton M, Maundrell K (1995). Activation of phospholipase C gamma in Schizosaccharomyces pombe by coexpression of receptor or nonreceptor tyrosine kinases.. Mol Cell Biol.

[26] Khare S, Bolt MJ, Wali RK, Skarosi SF, Roy HK (1997). 1,25 dihydroxyvitamin D3 stimulates phospholipase C-gamma in rat colonocytes: role of c-Src in PLC-gamma activation.. J Clin Invest.

[27] Townley IK, Schuyler E, Parker-Gur M, Foltz KR (2009). Expression of multiple Src family kinases in sea urchin eggs and their function in Ca2+ release at fertilization.. Dev Biol.

[28] Boggon TJ, Eck MJ (2004). Structure and regulation of Src family kinases.. Oncogene.

[29] Okada M, Nada S, Yamanashi Y, Yamamoto T, Nakagawa H (1991). CSK: a protein-tyrosine kinase involved in regulation of src family kinases.. J Biol Chem.

[30] Luttrell DK, Luttrell LM (2004). Not so strange bedfellows: G-protein-coupled receptors and Src family kinases.. Oncogene.

[31] Byrne RD, Zhendre V, Larijani B, Poccia DL (2009). Nuclear envelope formation in vitro: a sea urchin egg cell-free system.. Methods Mol Biol.

[32] Foltz KR, Adams NL, Runft LL (2004). Echinoderm eggs and embryos: procurement and culture.. Methods Cell Biol.

[33] Giusti AF, O’Neill FJ, Yamasu K, Foltz KR, Jaffe LA (2003). Function of a sea urchin egg Src family kinase in initiating Ca2+ release at fertilization.. Dev Biol.

[34] Runft LL, Carroll DJ, Gillett J, Giusti AF, O’Neill FJ (2004). Identification of a starfish egg PLC-gamma that regulates Ca2+ release at fertilization.. Dev Biol.

[35] Alcor D, Calleja V, Larijani B (2009). Revealing signaling in single cells by single- and two-photon fluorescence lifetime imaging microscopy.. Methods Mol Biol.

[36] Calleja V, Alcor D, Laguerre M, Park J, Vojnovic B (2007). Intramolecular and intermolecular interactions of protein kinase B define its activation in vivo.. PLoS Biol.

[37] O’Neill FJ, Gillett J, Foltz KR (2004). Distinct roles for multiple Src family kinases at fertilization.. J Cell Sci.

[38] Stricker SA, Carroll DJ, Tsui WL (2010). Roles of Src family kinase signaling during fertilization and the first cell cycle in the marine protostome worm Cerebratulus.. Int J Dev Biol.

[39] Sharma D, Kinsey WH (2006). Fertilization triggers localized activation of Src-family protein kinases in the zebrafish egg.. Dev Biol.

[40] Collas P, Courvalin JC, Poccia D (1996). Targeting of membranes to sea urchin sperm chromatin is mediated by a lamin B receptor-like integral membrane protein.. J Cell Biol.

[41] Collas P, Poccia DL (1996). Conserved binding recognition elements of sperm chromatin, sperm lipophilic structures and nuclear envelope precursor vesicles.. Eur J Cell Biol.

[42] Collas P, Poccia DL (1995). Formation of the sea urchin male pronucleus in vitro: membrane- independent chromatin decondensation and nuclear envelope-dependent nuclear swelling.. Mol Reprod Dev.

[43] Byrne RD, Barona TM, Garnier M, Koster G, Katan M (2005). Nuclear envelope assembly is promoted by phosphoinositide-specific phospholipase C with selective recruitment of phosphatidylinositol-enriched membranes.. Biochem J.

[44] Cooper JA, King CS (1986). Dephosphorylation or antibody binding to the carboxy terminus stimulates pp60c-src.. Mol Cell Biol.

[45] Anderson DJ, Hetzer MW (2007). Nuclear envelope formation by chromatin-mediated reorganization of the endoplasmic reticulum.. Nat Cell Biol.

[46] Antonin W, Ellenberg J, Dultz E (2008). Nuclear pore complex assembly through the cell cycle: regulation and membrane organization.. FEBS Lett.

[47] Baur T, Ramadan K, Schlundt A, Kartenbeck J, Meyer HH (2007). NSF- and SNARE-mediated membrane fusion is required for nuclear envelope formation and completion of nuclear pore complex assembly in Xenopus laevis egg extracts.. J Cell Sci.

[48] Byrne RD, Larijani B, Poccia DL (2009). Tyrosine kinase regulation of nuclear envelope assembly.. Adv Enzyme Regul.

[49] Townley IK, Roux MM, Foltz KR (2006). Signal transduction at fertilization: the Ca2+ release pathway in echinoderms and other invertebrate deuterostomes.. Semin Cell Dev Biol.

[50] Bradham CA, Foltz KR, Beane WS, Arnone MI, Rizzo F (2006). The sea urchin kinome: a first look.. Dev Biol.

[51] Hetzer M, Bilbao-Cortes D, Walther TC, Gruss OJ, Mattaj IW (2000). GTP hydrolysis by Ran is required for nuclear envelope assembly.. Mol Cell.

[52] Stenmark H (2009). Rab GTPases as coordinators of vesicle traffic.. Nat Rev Mol Cell Biol.

[53] Conner S, Wessel GM (1998). rab3 mediates cortical granule exocytosis in the sea urchin egg.. Dev Biol.

[54] Conner SD, Wessel GM (2000). A rab3 homolog in sea urchin functions in cell division.. Faseb J.

[55] Ma YC, Huang J, Ali S, Lowry W, Huang XY (2000). Src tyrosine kinase is a novel direct effector of G proteins.. Cell.

[56] Voronina E, Wessel GM (2004). betagamma subunits of heterotrimeric G-proteins contribute to Ca2+ release at fertilization in the sea urchin.. J Cell Sci.

[57] Gould GW, Lippincott-Schwartz J (2009). New roles for endosomes: from vesicular carriers to multi-purpose platforms.. Nat Rev Mol Cell Biol.

