# Supplementary figures and images for: Dynamics of PLCγ and Src Family Kinase 1 Interactions during Nuclear Envelope Formation Revealed by FRET-FLIM
Source: PLoS One. 2012 Jul 24;7(7):e40669. doi: 10.1371/journal.pone.0040669 (PMC3404105; doi:10.1371/journal.pone.0040669)

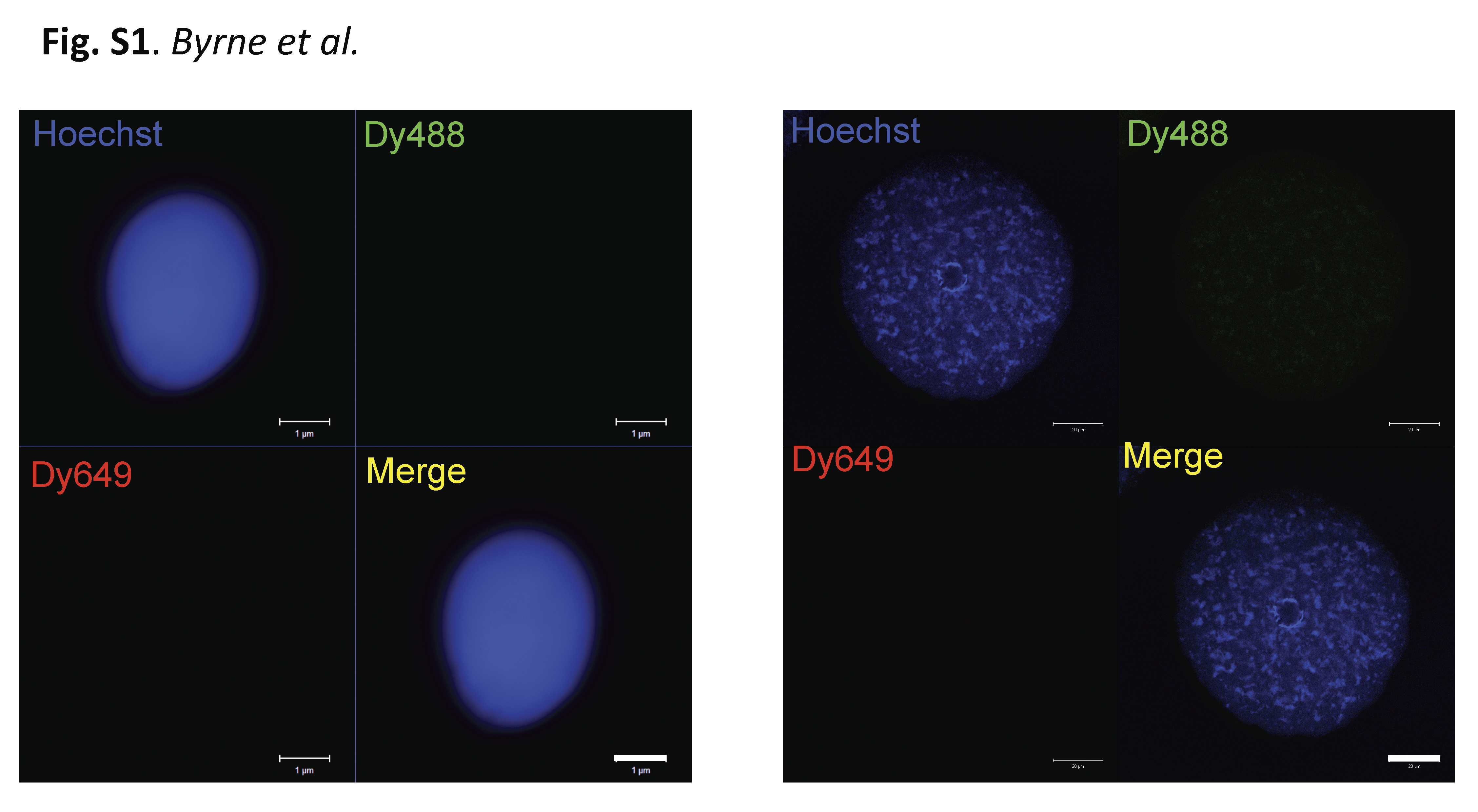

Supplement: Figure S1 — Decondensed sperm nuclei (left) or T0 (unfertilised) eggs (right) were prepared as described in the methods section, and stained with DyLight 488 and DyLight 649 alone (both at 1∶500) in the absence of primary antibodies. Samples were additionally stained with Hoechst 33342 and imaged by confocal microscopy. Note the blue channel of the egg image has been deliberately enhanced to show the dimensions of the egg. Scale bar 1 µm (nuclei) and 20 µm (egg). (TIFF) [file pone.0040669.s001.tiff]

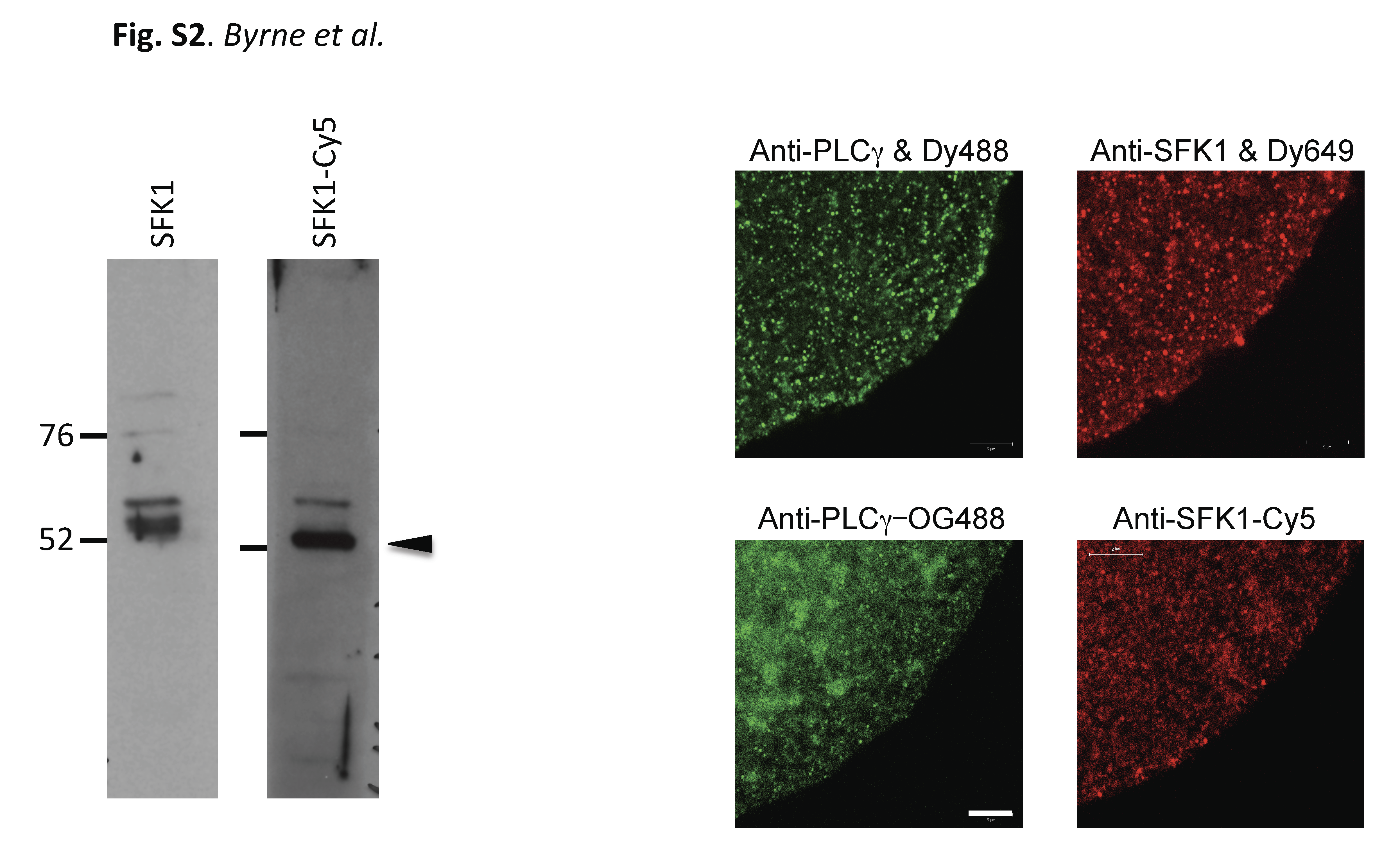

Supplement: Figure S2 — Left: Anti-SFK1 blot undertaken with antibodies in the absence and presence of Cy5 respectively. The antibody concentration was equal in both blots. Relevant Mr are indicated (×103). The arrow indicates the antigen of predicted size for SFK1. Right: T0 (unfertilised) eggs were prepared as described in the methods section, and stained with anti-PLCg plus a DyLight 488 secondary, anti-PLCγ conjugated to Oregon green 488, anti-SFK1 plus a DyLight 649 secondary or anti-SFK1 conjugated to Cy5. Samples were imaged by confocal microscopy. Conjugated antibodies continued to recognise vesicles in the egg cortex. As the FRET assay is a 2-site assay any non-specific signal/egg autofluoresence will not lead to a non-specific signal in our assay. Scale bar is 5 µm. (TIFF) [file pone.0040669.s002.tiff]

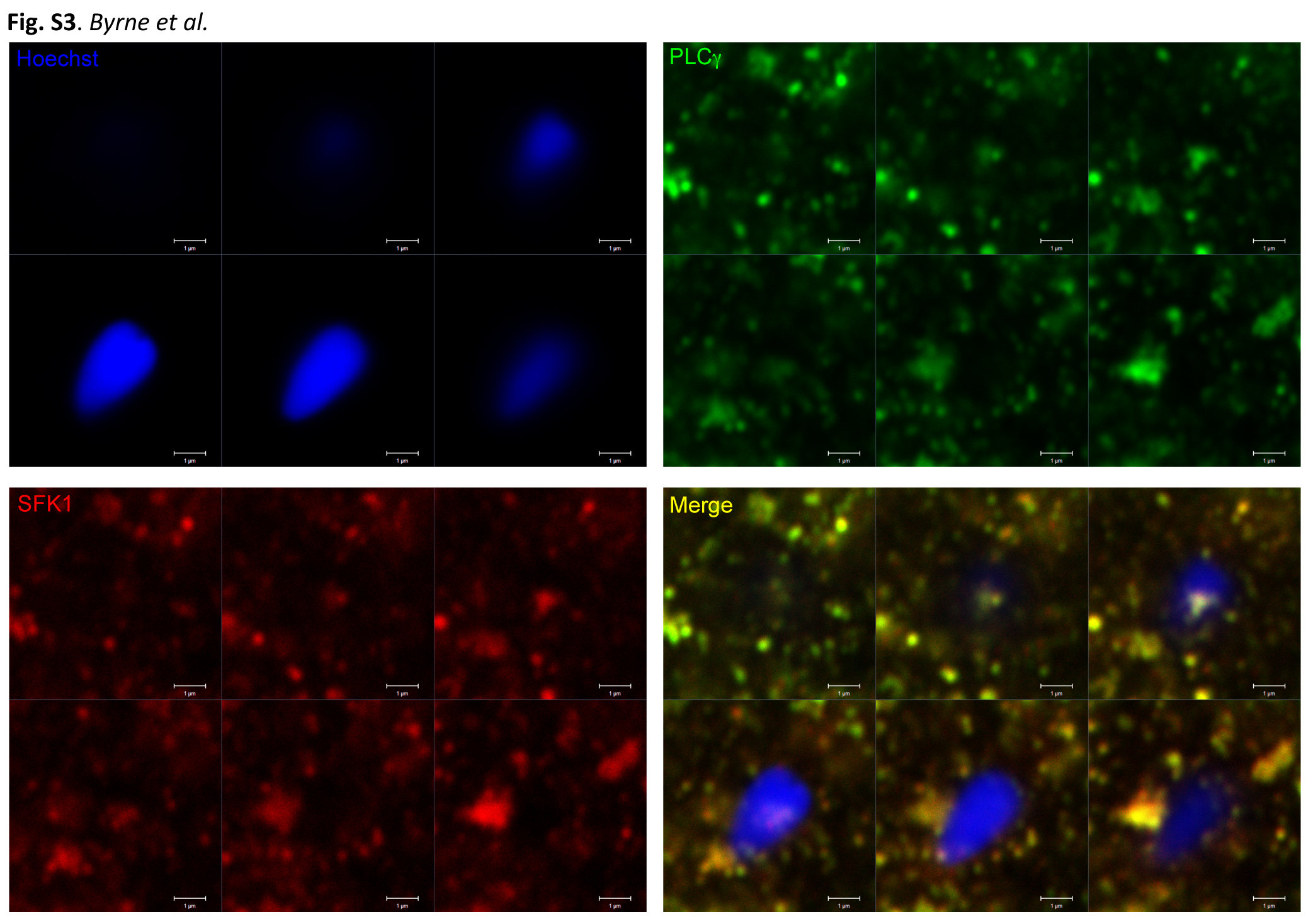

Supplement: Figure S3 — A T+5 egg fixed, and labelled with Hoechst 33342, anti-PLCg followed by a DyLight 488 secondary and anti-SFK1-Cy5 direct conjugate. The complete confocal z-series is shown, encompassing the sperm nucleus. Scale bar is 1 µm. (TIF) [file pone.0040669.s003.tif]

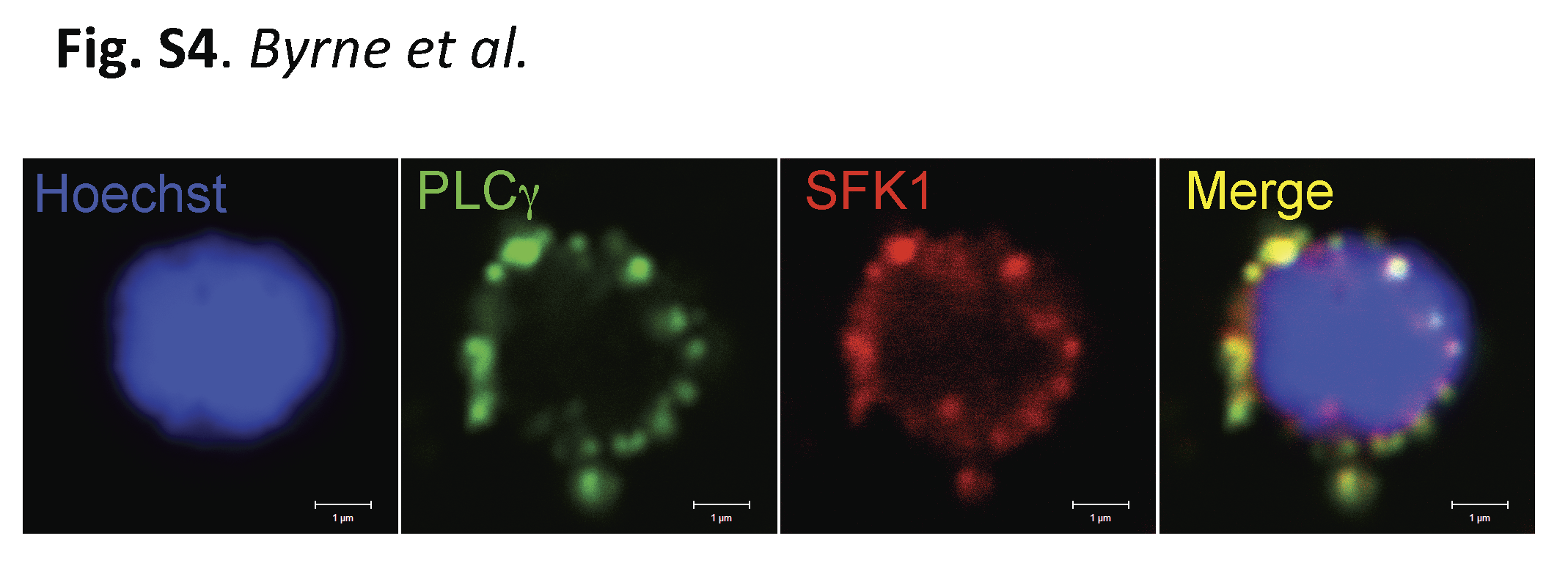

Supplement: Figure S4 — Demembranated sperm nuclei were decondensed in fertilised egg cytoplasmic extract in the presence of ATP-GS. Nuclei were fixed and labelled with Hoechst 33342 (blue), anti-PLCγ and anti-SFK1 directly conjugated to OG488 (green) and Alexa 546 (red) respectively. The images shown were obtained by confocal microscopy to confirm the recruitment of PLCγ and SFK1 to the NE could be detected with the reagents for the subsequent FRET experiments. The co-localisation of PLCγ and SFK1 further confirms the retention of the specificity of the conjugated antisera. Scale bar is 1 µm. (TIFF) [file pone.0040669.s004.tiff]

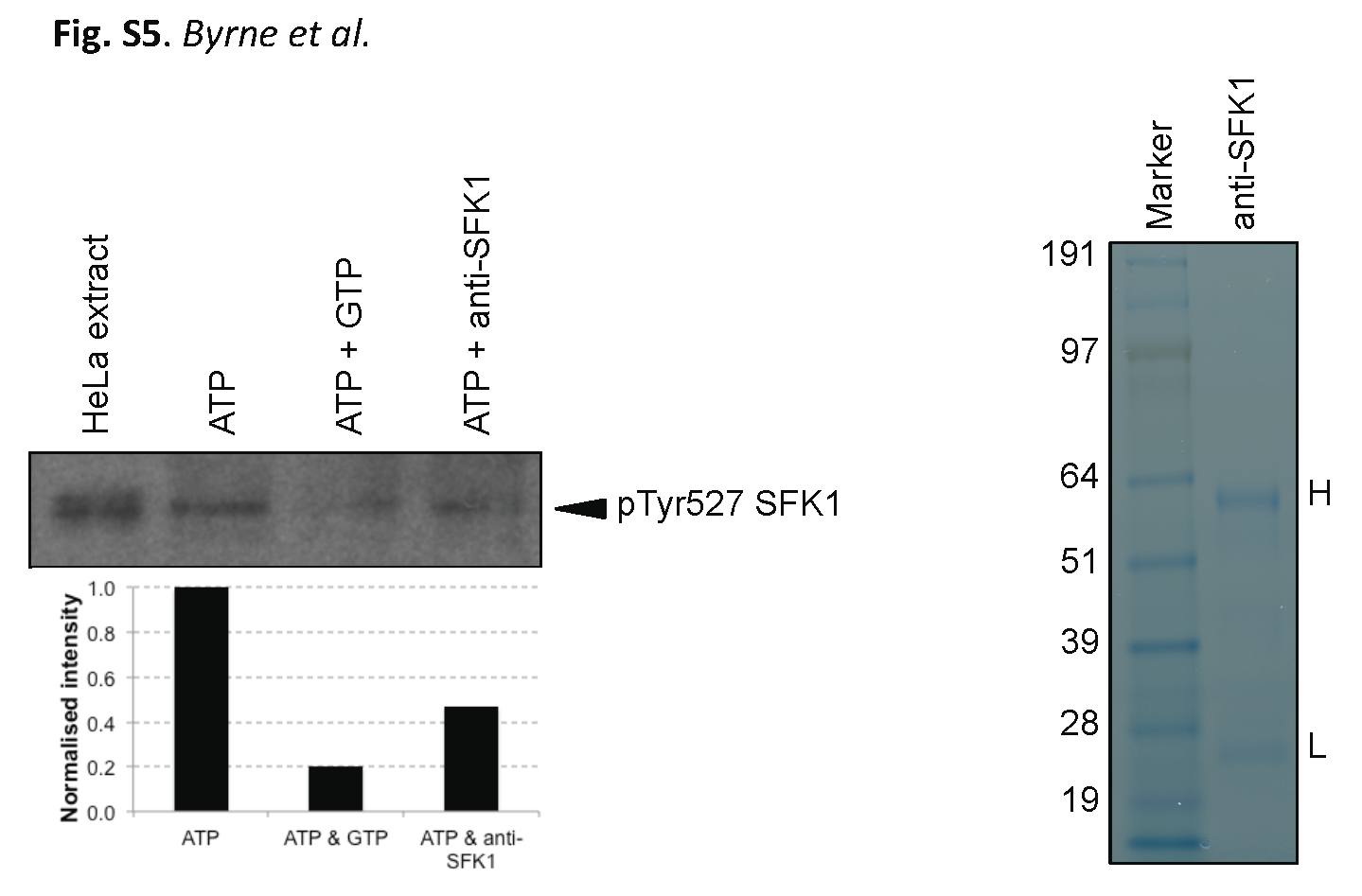

Supplement: Figure S5 — Left: in vitro NE formation assays were performed as described in the methods section. Membrane fusion events were induced with 1 mM GTP or 1 µg/ml anti-SFK1 antibody for 5 minutes. Nuclei were separated from unbound vesicles by centrifugation through a sucrose cushion, and nuclei pellets resuspended in 4× SDS buffer. The Tyr527 site of SFK1 was probed by western analysis with an anti-pTyr527 antibody, and band intensity quantified in Image J. Right: 3 µg anti-SFK1 was separated on a 4–12% pre-cast bis-tris gel and stained with colloidal coomassie blue stain (Thermo). The heavy (H) and light (L) chain of the affinity purified IgY were visualised. Note the IgY heavy chain is 70 kDa. Mr is denoted in kDa. (TIFF) [file pone.0040669.s005.tiff]
